# Drug Resistance-Associated Mutations in *ERG11* of Multidrug-Resistant *Candida auris* in a Tertiary Care Hospital of Eastern Saudi Arabia

**DOI:** 10.3390/jof7010018

**Published:** 2020-12-31

**Authors:** Reem AlJindan, Doaa M. AlEraky, Nehal Mahmoud, Baha Abdalhamid, Mashael Almustafa, Sayed AbdulAzeez, J. Francis Borgio

**Affiliations:** 1 Department of Microbiology, College of Medicine, Imam Abdulrahman Bin Faisal University, Dammam 40017, Saudi Arabia; raljindan@iau.edu.sa (R.A.); nmhosin@iau.edu.sa (N.M.); 2 Department of Biomedical Dental Science, Microbiology and Immunology Division, College of Dentistry, Imam Abdulrahman Bin Faisal University, Dammam 40344, Saudi Arabia; 3 Department of Pathology and Microbiology, University of Nebraska Medical Center, Omaha, NE 68022, USA; babdalhamid@unmc.edu; 4 Department of Pharmacy, King Fahd Hospital, Imam Abdulrahman Bin Faisal University, Alkhobar 31952, Saudi Arabia; mmustafa@iau.edu.sa; 5 Department of Genetic Research, Institute for Research and Medical Consultations (IRMC), Imam Abdulrahman Bin Faisal University, Dammam 31441, Saudi Arabia; asayed@iau.edu.sa (S.A.); fbalexander@iau.edu.sa (J.F.B.); 6 Department of Epidemic Diseases Research, Institute for Research and Medical Consultations (IRMC), Imam Abdulrahman Bin Faisal University, Dammam 31441, Saudi Arabia

**Keywords:** *Candida auris*, nosocomial infections, emerging pathogen, resistance genes, Saudi Arabia

## Abstract

*Candida auris* is an emerging multi-drug resistant pathogen with high mortality rate; nosocomial infections have been reported worldwide, causing a major challenge for clinicians and microbiological laboratories. The study aims to describe new cases of *C. auris* and detect drug resistance-associated mutations of *C. auris* by the sequencing of *ERG11* and *FKS1* genes. A total of six specimens were collected from blood, urine, ear swab, and groin screening samples. Isolates were incubated for 48 h on Sabouraud Dextrose agar (SDA) at 42 °C, then confirmed by MALDI-TOF MS. Furthermore, antifungal susceptibility testing was performed using the Vitek 2 system to detect Minimum Inhibitory Concentrations (MICs) of six antifungals. Sequences of *18S rRNA* gene and ITS regions from isolates and phylogenetic analysis were performed. Gene sequencing was analysed to detect drug resistance-associated mutations by *FKS1* and *ERG11* genes sequencing. All *C. auris* isolates were confirmed by MALDI-TOF MS, and evolutionary analyses using sequences of *18S rRNA* gene and ITS region. Antifungal susceptibility testing showed that all isolates were resistant to fluconazole. Sequencing of *ERG11* and *FKS1* genes from the isolates revealed the presence of two (F132Y and K143R) drug resistance-associated mutations in *ERG11*, however, *FKS1* gene was devoid of mutations. The study sheds light on a public health threat of an emerging pathogen, and the hospital implemented strict contact screening and infection control precautions to prevent *C. auris* infection. Finally, there is a critical need to monitor the antifungal resistance in different geographical areas and implementation of efficient guidelines for treatment.

## 1. Introduction

*Candida auris* is an emerging pathogen that had been reported in the past decade as a rising threat and a challenging nosocomial infection [1]. *C. auris* tends to transmit rapidly from person to person, and persist on medical devices and on the surfaces of hospital areas [2,3].

It was first described in Japan from a culture of external ear canal in 2009, and within the last decade, *C. auris* was frequently isolated from the bloodstream, urinary, and respiratory tract. It has been reported from several countries in Africa, Asia, Europe, America, and the Middle East, with significant fatality rate [4].

The detection of *C. auris* is still a challenging dilemma for clinical laboratories because it is closely related to other *Candida species* such as, *C. haemulonii*, *C. duobushaemulonii,* and *C. lusitaniae* [5,6]. *C. auris* grows well on Sabouraud and chromogenic agar at 37 °C and 42 °C, and there are different efficient methods to identify *C. auris*, such as molecular techniques and MALDI-TOF MS [7,8].

Azoles, echinocandins, and amphotericin B are the three main classes of antifungal agents, the increasing fluconazole resistance could be explained by mutations in the *ERG11* gene encoding lanosterol 14-alpha-demethylase, which has an essential role in the ergosterol synthesis pathway [9]. In contrast, echinocandin resistance remains quite low in most *Candida species* except *Candida glabrata*, and it is linked to mutations of *FKS*, which is the gene encoding the catalytic subunits of the enzyme β-1,3-D-glucan synthase that target the drug [10]. Antifungal sensitivity of *C. auris* can be evaluated by using either microdilution or disk diffusion test. However, identification of *ERG11* and *FKS1* genes mutations would be an advanced screening method to detect potentially resistant strains [11].

The first case from Saudi Arabia were reported in 2018, followed by other cases of *C. auris* infection from different cities [12,13,14,15,16]. The aim of this study is to describe new cases of *C. auris* infection and to detect antifungal resistance genes, namely, *ERG11* and *FKS1*, in isolates of this global emerging pathogen.

## 2. Materials and Methods

### 2.1. Samples Collection and Microbial Identification

A total of six specimens were collected from five patients who were admitted to a tertiary care hospital in Al Khobar, Saudi Arabia. Specimens were collected from sterile and non-sterile sites including blood, urine, ear swab, and groin screening samples during the period from November 2018 to April 2019. Clinical microbiology testing was performed according to standard operative procedures in the hospital using different culture media. All clinical isolates were incubated for 48 h on Sabouraud Dextrose agar at 42 °C, then confirmed by MALDI-TOF MS, and the identification procedure was performed according to the manufacturer’s protocol and guidelines for yeast identification [17].

### 2.2. Antifungal Susceptibility Testing

In vitro AFST was performed using the Vitek 2 system (AST-YS08 card: bioMérieux, Hazelwood, MO, USA). Minimum Inhibitory Concentrations (MIC) of 6 antifungals were tested as per the manufacturer’s instructions; amphotericin B, fluconazole, voriconazole, caspofungin, micafungin, and Flucytosine. In each test, two reference strains, *Candida parapsilosis* ATCC 22019 and *Candida krusei* ATCC 6258, were included as control strains. The interpretation of the results was based on the following MIC breakpoints for *C. auris* published by the CDC: Amphotericin B, 2 µg/mL; fluconazole, 32 µg/mL; voriconazole, not available; caspofungin, 2 µg/mL; and micafungin, 4 µg/mL; and Flucytosine ≤ 1 μg/mL.

### 2.3. DNA Extraction and Sequencing of 18S rRNA Gene

Total genomic DNA from isolates, CA1, CA3, CA4, CA5, CA7, and CA8 were extracted as per the manufacturer’s instruction using Qiagen’s Yeast/Bact. Kit (Gentra Puregene Yeast/Bact. Kit, Qiagen, Hilden, Germany). The *18S rRNA* gene (1655 bp) of all the strains was amplified using 18SrRNAF (5′-GCTTAATTTGACTCAACACGGGA-3′) and 18SrRNAR (5′-AGCTATCAATCTGTCAATCCTGTC-3′) primers, (MoleQule-On, Auckland, New Zealand) at annealing temperature at 61.8 °C using absolute master mix (MoleQule-On, Auckland, New Zealand) in T-Professional thermocycler (Biometra, Göttingen, Germany) for 35 cycles.

The PCR amplicons were purified using QIAquick PCR Purification Kit (Qiagen, Germany) after visualized the product using 2% agarose gel. The purified amplicons were sequenced using 3500 genetic analysers (Applied Biosystems, Forster City, CA, USA) with the forward and reverse primers used for the amplification using Big Dye^®^ Terminator v3.1 Cycle Sequencing Kit (Applied Biosystems, Forster City, CA, USA).

### 2.4. Molecular Identification

The *18S rRNA* gene sequence from isolates CA1, CA3, CA4, CA5, CA7, and CA8 were aligned and analysed using Basic Local Alignment Search Tool, PHYMYCO-DB, and FungiDB [18,19]. An evolutionary relationship of samples was constructed using the Maximum Likelihood method based on the Tamura-Nei model in MEGA7 software package with bootstrap consensus tree from 500 replicates [20,21,22].

### 2.5. Sequencing of ITS Region

Primers were designed for the amplification of the internal transcribed spacer 1 (*ITS1*), 5.8S ribosomal RNA, and internal transcribed spacer 2 (*ITS2*); forward primer: ITSbF 5′-AGGAATTCCTAGTAAGCGCAAGT-3′ and reverse primer: ITSbR 5′-ATTTACCACCCACTTAGAGCT-3′ (Integrated DNA technologies, Coralville, IA, United States). ITS region was amplified at 57.5 °C annealing temperature using absolute master mix (MoleQule-On, Auckland, New Zealand) in T-Professional thermocycler (Biometra, Göttingen, Germany) for 35 cycles. Amplicons were visualized using 2% agarose gel and documented. The amplified ITS region amplicons (840 bp) were purified and sequenced using 3500 genetic analysers (Applied Biosystems, Forster City, CA, USA) with the forward primers (ITSbF) using Big Dye^®^ Terminator v3.1 Cycle Sequencing Kit (Applied Biosystems, Forster City, CA, USA). The sequences were analysed using BLASTn and UNITE advanced analyses.

### 2.6. Sequencing of Resistance-Associated Mutations

Primers for the *FKS1* and *ERG11* genes were designed and used for the study. *FKS1* gene was amplified using forward (FKS1aF 5′ATGTCTTACGATAACAATCACAACTAC-3′), and reverse primers (FKS1aR 5′-AGTAAGATTCGGCCAACTTAGCAG-3′) (MoleQule-On, Auckland, New Zealand) with the 1900 bp amplicon. *ERG11* gene was amplified using the forward (ERG11aF 5′- ATGGCCTTGAAGGACTGCATCGT-3′) and reverse primers (ERG11aR 5′-TTAGTAAACACAAGTCTCTCTTTTCTCCCA-3′) (MoleQule-On, Auckland, New Zealand) with the 1575 bp amplicon. *FKS1* and *ERG11* genes were amplified at 59.4 °C and 62.2 °C annealing temperature, respectively, using absolute master mix (MoleQule-On, New Zealand) in T-Professional thermocycler (Biometra, Göttingen, Germany) for 35 cycles. Amplicons were visualized using 2% agarose gel and documented. The amplified *FKS1* and *ERG11* gene amplicons were purified and sequenced using 3500 genetic analysers (Applied Biosystems, Forster City, CA, USA) with the forward and reverse primers (FKS1aF & FKS1aR for FKS1; ERG11aF and ERG11aR for *ERG11*) separately using Big Dye^®^ Terminator v3.1 Cycle Sequencing Kit (Applied Biosystems, Forster City, CA, USA). The sequences were analysed using Mutation Surveyor with GenBank: KY410388.1 and GenBank: NW_021640162.1 as reference sequences for *ERG11* and *FKS1,* respectively.

## 3. Results

### 3.1. Culture Identification and Antifungal Susceptibility Testing

All specimens were collected from Saudi male patients with diabetic and different comorbidity diseases. Clinical samples were isolated on culture media, then confirmed by MALDI-TOF MS analysis.

Patients presented with signs of infection including hematuria, sepsis, decompensated heart failure secondary to hospital acquired pneumonia, and ear discharge. Meropenem was prescribed before and after isolation of *C. auris* in the first two patients in Table 1. The fourth patient received tazocin, cefazoline, and ceftazidime before and after isolation. Other antimicrobial agents including tri-sulfa, and levofloxacin were administrated before isolation *C auris.* Then, after isolation, patients received colistin, and tigecycline. Antifungal drugs such as: Amphotericin B, caspofungin, and anidulafungin were prescribed for patients with invasive infections. With respect to the prognosis, patients were critically ill and have already passed away due to serious co-morbidity.

Interpretation of antifungal susceptibility results showed that *C. auris* isolates were resistant to fluconazole with MICs ranged from 8 to 32 μg/mL. However, voriconazole with MIC, 0.5 μg/mL showed 3 (50%) resistant isolates, and the others (50%) were intermediate. Furthermore, 50% of *C. auris* isolates were resistant to Amphotericin B with MICs ranged from 8 to 32 μg/mL. Finally, caspofungin, micafungin, and flucytosine showed excellent activity with the tested isolates. Unfortunately, three patients died almost one month after detection of *C. auris* (Table 1).

### 3.2. ITS and 18S rRNA Sequencing

The sequences of internal transcribed spacer 1 (*ITS1*), 5.8S ribosomal RNA, and internal transcribed spacer 2 (*ITS2*) from all the samples were analysed using BLASTn and UNITE advanced analyses, and the results confirmed that all the strains are *C. auris,* as identified through *18S rRNA* gene. Sequences of ITS region were submitted to GenBank, accession number: CA1: MW039128.1; CA3: MW039130.1; CA4: MW039131.1; CA5: MW039132.1; CA7: MW039134.1; and CA8: MW039135.1.

**Figure 1:**
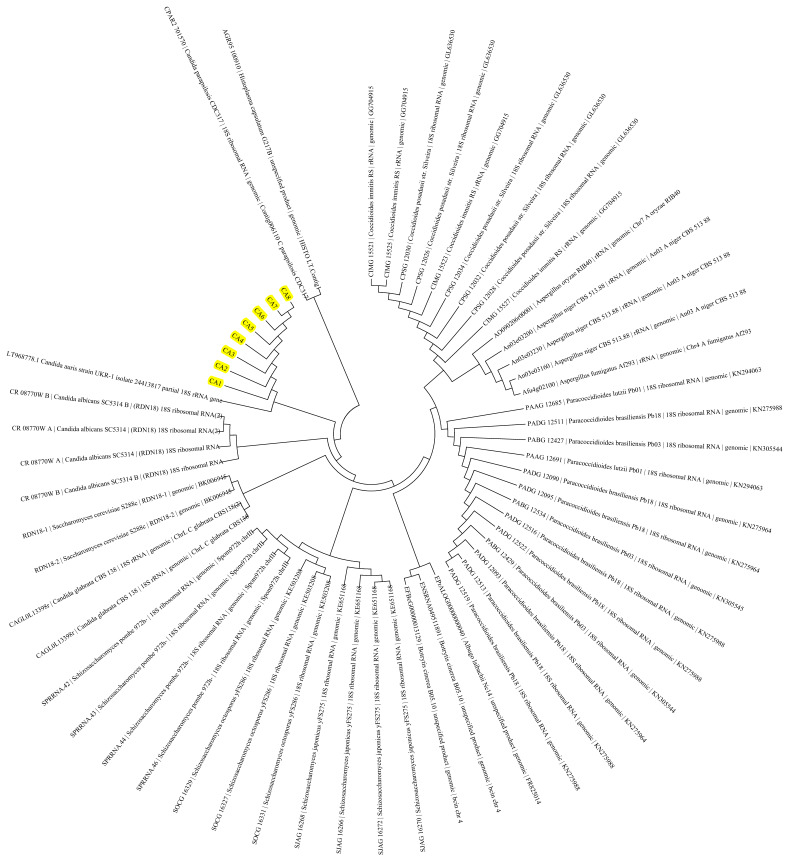
Molecular Phylogenetic analysis of the 6 isolates (CA1, CA3, CA4, CA5, CA7, and CA8) of *Candida auris* by Maximum Likelihood method. Six isolates of *Candida auris* and internal positive controls are highlighted yellow.

The evolutionary history was inferred by using the Maximum Likelihood method based on the Tamura-Nei model [22]. The bootstrap consensus tree inferred from 500 replicates is taken to represent the evolutionary history of the taxa analysed [20]. Branches corresponding to partitions reproduced in less than 50% bootstrap replicates are collapsed. Initial tree(s) for the heuristic search were obtained automatically by applying Neighbor-Join and BioNJ algorithms to a matrix of pairwise distances estimated using the Maximum Composite Likelihood (MCL) approach, and then selecting the topology with superior log likelihood value. The analysis involved 58 nucleotide sequences. Codon positions included were 1st + 2nd + 3rd + Noncoding. All positions containing gaps and missing data were eliminated. Evolutionary analyses were conducted in MEGA7 [21]. There were an overall of 333 positions in the final dataset, and the tree with the highest log likelihood (−1643.31) was demonstrated (Figure 1). Evolutionary analysis of the standard sequences of *C. auris* and the six isolates indicated no genetic divergence among the six isolates on *18S rRNA* gene and ITS region. However, whole genome comparisons are needed to confirm complete sequence divergences.

### 3.3. Resistance Genes Mutations

All samples were successfully amplified for both *ERG11* (1575 bp) and *FKS1* (1900 bp) genes. Sequences of *18S rRNA* gene were submitted to GenBank, accession number: CA1: MN658527.1; CA3: MN658529.1; CA4: MN658530.1; CA5: MN658531.1; CA7: MN658533.1; and CA8: MN658534.1.

**Figure 2:**
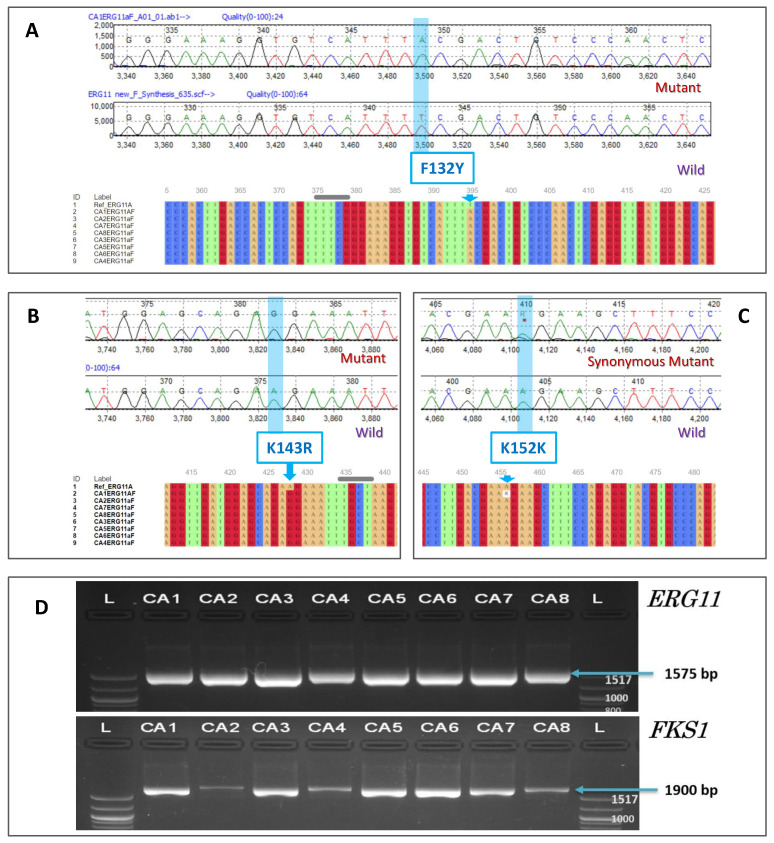
Resistance associated mutations in *ERG11* gene. Reference sequence used for analysis: GenBank: KY410388.1. (**A**): Electropherogram of F132Y mutant. Bottom: Multiple sequence alignment of region with T > A. (**B**): Electropherogram of K143R mutant. Bottom: Multiple sequence alignment of region with A > G. (**C**): Electropherogram of K152K mutant. Bottom: Multiple sequence alignment of region with A > G (AAA > AAG). (**D**): The *ERG11* (Lanosterol 14-alpha demethylase) and *FKS1* (1,3-beta-glucan synthase component) gene PCR amplification from *Candida auris*. L: 100 bp ladder; CA1, CA3, CA4, CA5, CA7, and CA8: Isolates of *Candida auris.* CA2 (GenBank: MK910118.1) and CA6 (GenBank: MK910117.1): Internal positive controls of *Candida auris*.

The amplicons were sequenced, and the sequences were analysed for the identification of the drug resistance-associated mutations in the *ERG11* and *FKS1* genes from the 6 isolates (CA1, CA3, CA4, CA5, CA7, and CA8) of *Candida auris.* The analysis revealed the presence of two (F132Y and K143R) drug resistance-associated mutations in *ERG11* gene. Furthermore, a synonymous heterozygous K152K mutation was observed in a strain. However, no mutations were detected in the *FKS1* genes (Table 2; Figure 2).

## 4. Discussion

*Candida auris* is the first fungal considered as a public health threat, as it can spread easily among patients in hospitals and may cause serious diseases [23]. High risk factors associated with *C. auris* infections include using broad spectrum antibiotics, catheters, ICU admission [6,24], and diabetic patients, and most infections arise two to seven weeks after admission. *C. auris* infection is correlated with higher resistance to fluconazole and moderately low resistance to echinocandins, therefore echinocandin is recommended as an empirical therapy before antifungal susceptibility testing of collected strains [4].

According to a study that reported the first hospital outbreak of *C. auris* in a European hospital, most of clinical manifestations of *C. auris* included colonization followed by candidemia, wound infections, urinary catheter infections, and others [25].

Guidelines of the CDC and recent publications stated that invasive infections may develop after colonization, and prophylactic antifungal treatment should be considered in case of a patient colonized with *C. auris* subsequently deteriorates [26,27,28]. In the present study, these six isolates were the only cases during the time period, 90% of the patients were diabetics and using broad spectrum antibiotics, with an age range from 62 to 85, except one patient, who was 26 years old. Caspofungin was prescribed and the hospital instituted strict contact screening and infection control precautions according to CDC guidelines [4,29].

In Saudi Arabia, the first two cases of *C. auris* were detected one to three months after the patients’ admission to hospitals, and echinocandins had been used for successful treatment [12,13]. Four more cases were reported, where 90% of the isolates were misidentified as *C. haemulonii*, and in the same year, one case was confirmed via MALDI-TOF MS [14,15]. Recently, a study reported a high mortality rate of seven patients with *C. auris* infection [16].

Present antifungal susceptibility results concur with a study in Kuwait, which reported multidrug resistance and high mortality rate among cases of *C. auris* infections [30]. Additionally, two studies in Oman reported of *C. auris* occurrence at different hospitals, isolates were highly resistant to fluconazole and the onset of infection after hospitalization ranged from one to two months [31,32].

Sequencing results showed that drug resistance-associated mutations F132Y and K143R were identified in all isolates. These findings concur with a study in 2018, which concluded that F132Y and K143R mutations could be initial markers for azole resistance in South Asian and South American clade [33].

A multi-centre study in India showed that 41% of isolates were resistant to two classes of antifungal agents and numerous *ERG11* mutations have been detected in *C. auris* isolates from several geographic areas [34]. MALDI-TOF MS and gene sequencing are advanced and expensive techniques. Thus, there is a critical necessity to develop cost-effective techniques for the detection of this emerging pathogen in underdeveloped countries [35]. Rapid detection of *ERG11* and *FKS1* has the potential to overcome the deficiencies of existing Minimum Inhibitory Concentration (MIC) to detect azole and echinocandin resistant *C. auris* [11]. Mutations of *ERG11* (F132Y and K143R) in *C. auris* were associated with to increased resistance to fluconazole [36].

The limitations of this study include the identification of the cause of death precisely, as those patients had comorbidity diseases, and only one isolate was obtained from a sterile site (bloodstream). Isolation of *C. auris* from groin and ear canal may merely reflect colonization rather than infection.

Three salient features of this study, descripting new cases of *C. auris* from different sites confirmed by MALDI TOF MS, ribosomal, ITS sequencing, and developing the phylogenetic analysis of the isolates, and sequence of *ERG11* and *FKS1* genes among *C. auris* isolates clearly confer drug resistant characteristics of the isolates.

Further studies are crucial to analyse Whole Genome Sequence (WGS) of *C. auris* isolates to discover the mechanism of resistance and potential effective treatment for this emerging pathogen.

## Figures and Tables

**Table 1:** MICs (Minimum Inhibitory Concentrations) of tested antifungal agents for six *Candida auris* isolated from five patients with comorbidity diseases.

Antifungal Agent	Isolates					
	CA1	CA3	CA4	CA5	CA7	CA8
Sex	Male	Male	Male	Male		Male
Age	62	85	26	77		68
Medical condition	DM, HTN, CAD, right MCA stroke	DM, HTN, COPD, CVA	Brain stem haemorrhage	DM, HTN, chronic anaemia, ischaemic stroke, subarachnoid haemorrhage		DM, HTN, post cardiac valve replacement
Specimen	Urine	Urine	Groin	Blood	Urine	Ear swab
Amphotericin B	0.5	8	0.5	8	8	0.5
Fluconazole	16	32	32	32	32	8
Voriconazole	0.5	0.5	0.5	R–1	1	≥8
Caspofungin	0.25	0.25	0.25	0.25	0.25	0.25
Micafungin	≤0.06	≤0.06	≤0.06	≤0.06	≤0.06	≤0.06
Flucytosine	≤1	S–≤ 1	S–≤ 1	S–≤ 1	S–≤ 1	S–≤ 1

DM: Diabetes, HTN: Hypertension, CAD: Chronic artery disease, MCA: Middle cerebral artery, COPD: Chronic obstructive pulmonary disease, CVA: Cerebrovascular stroke.

**Table 2:** List of resistance-associated mutations in *ERG11* and *FKS1* genes in the *C. auris* isolates.

Gene	Mutation	CA1	CA3	CA4	CA5	CA7	CA8
*ERG11*	F132Y	+	+	+	+	+	+
*ERG11*	K143R	+	+	+	+	+	+
*ERG11*	K152K	+	-	-	-	-	-
*FKS1*	-	-	-	-	-	-	-

Reference sequence used for *ERG11* and *FKS1* are GenBank: KY410388.1 and GenBank: NW_021640162.1, respectively. -: No substitutions of nucleotides observed. +: Substitutions of nucleotides observed.

## Data Availability

The data presented in this study are available on request from the corresponding author. The data are not publicly available due to privacy.

## Abbreviations

The following abbreviations are used in this manuscript: *C. auris: Candida auris*; MALDI TOF MS: Matrix-Assisted Laser Desorption/Ionization-Time of Flight mass spectrometry; PCR: Polymerase Chain Reaction; IPP: Infection Prevention Program; CDC: Centers for Disease Control and prevention; ITS: Internal transcribed spacer; WGS: Whole Genome Sequencing.
